# Factors associated with modern contraceptive use among young and older women in Uganda; a comparative analysis

**DOI:** 10.1186/1471-2458-14-926

**Published:** 2014-09-08

**Authors:** John Bosco Asiimwe, Patricia Ndugga, John Mushomi, James Patrick Manyenye Ntozi

**Affiliations:** Department of Planning and Applied Statistics, School of Statistics and Planning, College of Business and Management Sciences, Makerere University, Kampala, Uganda; Department of Population Studies, School of Statistics and Planning, College of Business and Management Sciences, Makerere University, Kampala, Uganda

**Keywords:** Family planning, Modern contraception, DHS, Uganda, Young women, Older women, Age differences, Media messages, Village health teams

## Abstract

**Background:**

Much of the research literature about the use of family planning generalizes contraceptive use among all women, using age as a covariate. In Uganda, a country with divergent trends in modern family planning use, this study was set to explore whether or not the predictors of contraceptive use differ by age. This was assessed by using data from the 2011 Uganda Demographic and Health Survey (UDHS).

**Methods:**

We restricted the sample from each round to fecund, non-pregnant married women age 15–34 who were sexually active within one year prior to the survey, resulting in a sample of 2,814 women. We used logistic regression with age variable used as an interaction term to model the relationship between selected independent variables and the outcome variable (modern contraception use) for each group of women.

**Results:**

We found that the key factors associated with use of modern contraceptives varied among young and older married women age 15–24 and 25–34 respectively. Results showed that perception on distance to health facility, listening to radio and geographical differences exhibited significant variability in contraceptive use among the young and the older women. Other key factors that were important for both age groups in explaining contraceptive use were; desire to have children after two years and education level.

**Conclusions:**

Addressing contraceptive use among old and young women in Uganda requires concerted efforts that target such women to address the socio economic barriers that exist. There is need for increased access of family planning service to the population through strengthening the use of Village Health Teams (VHTs) whose service is currently limited in coverage (MoH, 2009). Given the variation in contraceptive use between the two age groups, our findings further suggest that there is need for variability in media targeting among the young and the older women categories for improved use of modern contraceptives, for instance using alternative media strategies to reach the young women. Family planning policies should also be tailored to address the specific needs of different age groups of women with varied geographical locations.

## Background

For countries that have achieved Millennium Development Goal 5 on improving maternal health, meeting women’s contraceptive needs has played an important role. MDG 5a aims to reduce the maternal mortality ratio by three-quarters between 1990 and 2015, and MDG 5b aims to achieve universal access to reproductive health, including family planning [1].

Satisfying the unmet need for family planning alone could cut the number of maternal deaths by almost a third [2]. However, an estimated 215 million women who would prefer to delay or avoid pregnancy continue to lack access to safe and effective contraception [2]. Thus along with providing skilled maternal care, offering family planning is crucial to averting maternal deaths.

Although many United Nations member countries, particularly those in the developed world, have strong family planning programs, this is not the case in sub-Saharan Africa, where despite a rise in contraceptive prevalence, many women continue to have unmet need for contraception [3, 4]. The resultant high fertility is associated with high levels of maternal mortality, especially among the poorest communities.

In Uganda, the maternal mortality ratio was estimated to be much higher than the worldwide average in 2011, at 438 per 100,000 births [5]. An estimated one-third of women who give birth in developing countries are below age 20, which exposes them to greater risk of illness and death related to maternal causes [6].

Furthermore, in Uganda as in many other countries, major [7] factors associated with contraceptive use are women’s age, education, and socioeconomic status. Ugandan women who are more educated and wealthier are more likely to use contraception compared with illiterate and less wealthy women [8]. Similarly, women who use contraceptives tend to have a better quality of life, higher social status, and greater autonomy. Contraceptive use has the power to reduce fertility considerably and ultimately to improve maternal and child health [7].

Uganda has a young population (52% are below age 15, and 17% are age 15–24) and a high total fertility rate (TFR), at 6.2 children per woman [5]. As this large cohort of young people enters the childbearing years, their reproductive behavior will determine the growth and size of Uganda’s population for decades to come. Uganda still struggles with a low contraceptive prevalence rate (CPR) of 30%, which is lower than that of her neighbors, Kenya, Rwanda, and Tanzania, which had a CPR of 46%, 52%, and 34%, respectively, at the time of their last surveys [9].

Modern contraceptive use (MCU) among young women, whether married or unmarried, involves a lot of experimentation and is inconsistent [10]. Additionally, young women face many barriers which hinder utilization of family planning services and these include; fear of side effects, cost, and lack of knowledge [10]. In the Ugandan context, only 24% of all Ugandan sexually active are married women [8]. Whereas age at first marriage has generally increased around the world, several parts of sub-Saharan Africa are struggling with a significant proportion of girls being married off before their 18th birthday [11]. Early marriage exposes these women to frequent and unprotected sexual intercourse, which can lead to early and risky first birth [12, 13]. In Uganda, the median age at first marriage is 17.9 years, and young women are expected to prove their fertility soon after marriage [6]. In addition, these women have a limited chance to space their births, since contraceptive use within marriage is not expected.

Understanding the key factors influencing contraceptive use among young and older married women who are at risk of unwanted pregnancies is key to the development of effective family planning programs. This study focused on married women (age 15–34) because, in Uganda, the majority of births occur within marriage. Given the context of high fertility in Uganda, this study assessed whether or not the predictors of contraceptive use differ by age.

## Methods

### Data used

The paper used secondary data of 2011 Uganda Demographic and Health Surveys (UDHS) obtained with permission from the measureDHS website. In this survey, two-stage cluster sampling was used to generate a nationally representative sample of households. The first stage involved selecting clusters from sampling frames used in recent nationwide surveys, followed by the second stage, which selected households in each cluster. Stratification of urban and rural areas was taken into account. A total of 8,674 women were interviewed.

### Measurement of variables and statistical methods

The statistical analysis focused only on married women age 15–34 years who were sexually active in the year before the survey and who were not pregnant or infecund, comprising a weighted sample size of 2,814. In order to ensure representativeness across the country and to correct for non-response, data used were weighted and took into consideration the complex survey design in the analyses, using the SVY command in Stata.

The main variable of interest (dependent variable) was MCU, which is binary in nature (non-use or use). Modern methods of family planning refer to safe, effective and legal methods to prevent pregnancy such as the pill, condoms, injectables, and the Intra-Uterine Device (IUD). The explanatory (independent) variables in the study are women’s education, wealth index, administrative region of the country, exposure to family planning messages or listens to a radio, television, or newspapers in the past few months prior to when the survey was carried out, desire for children, women’s empowerment, age at first marriage, ability to refuse sex, and visited health facility in the past 12 months.

In the final model, only variables that passed the multicollinearity test were included, with none of the variables having a VIF greater than 3. Empowerment was thought to be an important variable in the analysis. As mentioned above, the survey asked women about who makes decisions about four areas: major household purchases, daily household purchases, visits to family relatives, and own health care. The possible answers to these questions were: woman alone, woman with husband, husband alone, or another person altogether. If the woman indicated that she had a say in a decision, whether by herself or jointly with her husband, she received a score of 1. Based on the responses to these four questions, an empowerment index was constructed. Respondents were classified into three groups: low (0), medium (1–2), and high (3–4), depending on the number of decisions in which the woman said that she had a say.

A woman’s ability to refuse sex with her husband, also asked in the surveys, was considered separately from the empowerment index, both because it is more directly related to use of family planning and because the measure is constructed differently than the measure of empowerment. That is, women were asked whether they could refuse sex or not.

Family planning use in the highest wealth quintile is nearly double that of the next quintile, leading us to hypothesize that the top quintile should be considered separately from other groups. Therefore, wealth scores were collapsed into three groups: bottom 40%, middle 40% and top 20%.

The education variable was divided into six categories—no education, lower primary (primary one to three), upper primary (primary four to six), completed primary, secondary and post secondary based on the highest grade of schooling the respondent had attended. Attending school rather than obtaining a degree was used as a metric of education so that teenage women attending school or who might soon attend secondary school would not be excluded from those over age 18, who have had a chance to complete their degree.

**Figure 1:**
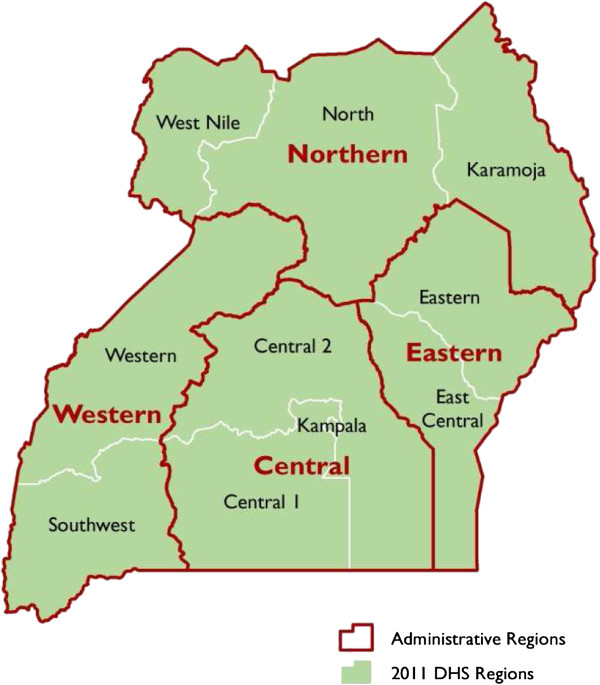
**Regional Groupings, using UDHS 2011 data.**

The regions used in DHS reports were regrouped into the ten Ugandan administrative regions for the purpose of the analysis. Figure 1 shows classification of regions in 2011. The regions were; Kampala, Central 1, Central 2, East Central, Eastern, Northern, Karamoja, West Nile, Western and South Western.

The paper starts with descriptive exploration of both dependent and independent variables. At the multivariate level, the relationship between selected variables of interest and the dependent variable (current use of modern contraception) was estimated using a multiple logistic regression model. Given that the core analytical strategy in this paper focuses on two age groups of women, young and older, an interactive term using the variable of age was used.

Dummy variables were automatically created using Stata software from select socioeconomic and demographic variables in the logistic regression model. Results were accepted at the 95% confidence level. The full regression model was also tested for goodness of fit using the Archer-Lemeshow goodness-of-fit test in Stata software. This model is based on the earlier Hosmer-Lemeshow test (1980) but adjusts for complex survey samples. The null hypothesis under the goodness of fit is that the model is a good fit, implying that probabilities greater than 0.05 using the 95% level of confidence were taken to be a good fit.

One major limitation of this dataset was heaping in some categories, for example education level. In this instance, women who had stopped at senior secondary level one were considered to be equal to those who had completed senior four. This categorization was necessary because women in such categories might exhibit similar literacy skills in terms of understanding family planning messages.

Another limitation of the study was that some desired aspects in the conceptual framework, for example experience of an abortion and woman’s HIV status, could not be adequately operationalized to allow analysis using the available dataset. Abortion data that is captured in the UDHS dataset does not stipulate whether they refer to spontaneous or induced abortion.

This study provides descriptive statistics about sexually active, fecund, non-pregnant married women age 15–24 (young) and age 25–34 (older). Table 1 shows the composition of women in each age group by different characteristics from the conceptual framework, including women’s education, wealth index, region of the country, and exposure to family planning messages. Statistically significant differences were tested using the chi-square test between the two age groups of women.

**Table 1:** **Percentage distribution of sexually active married women by age group and socio-economic characteristics (2011 UDHS)**

Background characteristics	Age group		
	15-24	25-34	Total
Education Attainment			
No education	5.0	14.3	10.9
Lower Primary (P1-P3)	17.4	25.7	22.5
Upper Primary (P4-P6)	32.1	20.6	25.0
Complete Primary	17.9	12.9	14.8
Secondary	26.3	20.0	22.4
Post Secondary	0.9	6.6	4.4
p-value	0.0000		
Wealth Index			
Lowest 40%	43.0	36.5	39.0
Middle 40%	34.5	36.5	35.7
Highest 20%	22.5	27.0	25.3
p-value	0.0130		
Administrative Region			
Kampala	8.3	9.0	8.8
Central1	10.1	10.5	10.4
Centra2	11.5	11.4	11.5
East Central	10.2	11.8	11.1
Eastern	20.2	12.4	15.4
Northern	9.1	9.5	9.3
Karamoja	2.7	3.3	3.1
West Nile	5.8	5.0	5.3
Western	12.3	13.9	13.3
South Western	9.9	13.1	11.9
p-value	0.0010		
Family Planning Messages			
Did not hear family planning message On radio, TV, or newspaper in past Few months	23.9	24.1	24.0
Head family planning message On radio, TV, or newspaper in past Few months	76.1	75.9	76.0
p-value	0.0158		
Listen to Radio			
Not listen to radio	17.8	15.4	16.3
Listen to radio	82.2	84.5	83.7
p-value	0.1864		
Desire for children			
Wants within 2 years	25.7	16.4	20.0
Wants after 2 years	72.4	78.0	75.8
Do not want any more	2.0	5.6	4.2
p-value	0.0000		
Age at first marriage in years			
<=12	4.1	4.1	4.1
13-17	56.0	48.8	51.5
18-24	40.0	42.6	41.6
25+	0.0	4.5	2.8
p-value	0.0000		
Can refuse sex			
No	13.4	13.7	13.6
Yes	86.6	86.3	86.4
p-value	0.8577		
Empowerment Index			
Low	22.3	17.0	19.0
Medium	40.0	35.7	37.3
High	37.8	47.4	43.7
p-value	0.0004		
Visited by family planning provider in last 12 months			
No	24.1	22.7	23.2
Yes	75.9	77.3	76.8
p-value	0.4879		
Average number of living children	1.7	3.7	2.9
Number of women (weighted)	1,076	1,738	2,814

## Results

### Descriptive statistics of the respondents

Results presented in Table 1 show that, younger women were better educated than women age 25–34. In regard to household wealth status, younger women seem to be more financially disadvantaged than older women in our sample.

The majority of women studied wanted to have the next child two years later. Results show that younger women showed stronger fertility desire than older women. About 26% of women age 15–24 wanted a child within two years compared with 16% among women age 25–34.

Younger women generally appear to be less empowered than older women. There was no significant difference between women’s ability to refuse sex among the young and older women. The percentages were similar for both age groups. Exposure to family planning message was also not significantly different between the young and the older women. In both age groups 76% of women reported to have been exposed to family planning message in the media (radio, television, newspaper) in the past few months preceding the survey. Listening to radio was quite high and was found not significantly differing among the young (82%) and the older women (85%).

**Figure 2:**
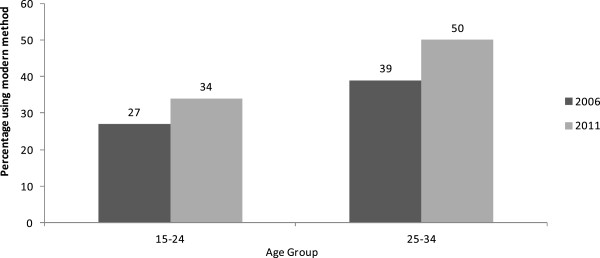
**Trend in use of modern contraception by married sexually active women age 15–24 and age 25–34 using 2011, UDHS Data.**

Figure 2 shows the proportion of married sexually active women using modern contraceptive methods by age group. In 2011, contraceptive use among sexually active women (excluding pregnant and infecund women) age 15–24 was lower, at 34%, than among women age 25–34, at 50%.

**Figure 3:**
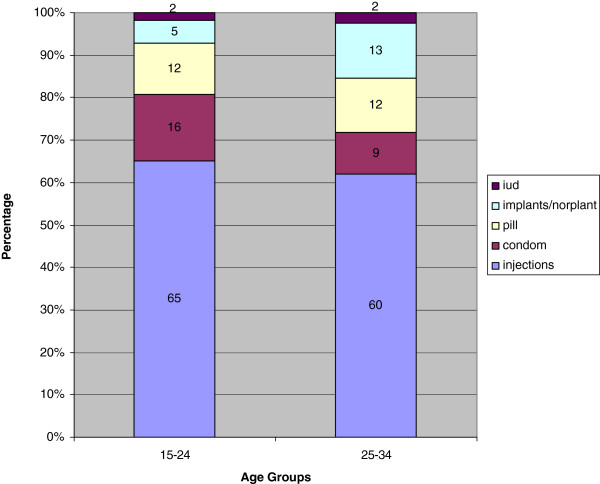
**Percentage distribution of contraceptive method mix, married women, using 2011, UDHS Data.**

Figure 3 shows the contraceptive method mix among sexually active married women by the year 2011. Injections were the predominantly used method among both the young and the older women. Figure 3 also shows that use of condoms and the pill was second to injections, though at relatively low levels.

This study also explored factors that might be associated with contraceptive use at a bivariate level of analysis, using the chi-square test to compare family planning use within each age group. Table 2 shows the percentage of women in each age group currently using a modern contraceptive method by selected characteristics of the women, including education, household wealth status, region, access to radio, exposure to family planning messages, fertility desire, age at first marriage, women’s ability to refuse sex, empowerment index, and contact with family planning health facilities.

**Table 2:** **Percentage distribution of married sexually active women currently using a modern method of contraception by socio-economic characteristics (UDHS 2011)**

Background characteristics	Age group	
	15-24	25-34
**Education Attainment**		
No education	15.5	22.6
Lower Primary (P1-P3)	17.5	33.6
Upper Primary (P4-P6)	25.5	32.7
Complete	21.6	33.7
Secondary	33.9	48.4
Post Secondary	68.1	49.3
p-value	0.0007	0.0000
**Wealth Index**		
Lowest 40%	19.1	25.8
Middle 40%	24.8	36.1
Highest 20%	38.7	49.1
p-value	0.0000	0.0000
**Administrative Region**		
Kampala	46.3	47.5
Central1	31.8	41.3
Central2	27.3	37.6
East Central	33.3	31.6
Eastern	17.8	34.1
Northern	16.3	39.8
Karamoja	15.9	15.8
West Nile	12.2	25.5
Western	31.1	37.6
South Western	18.6	31.7
p-value	0.0007	0.0250
**Family Planning Messages**		
Did not hear family planning message On radio, TV, or newspaper in past Few months	23.2	25.1
Heard family planning message On radio, TV, or newspaper in past Few months	26.2	39.3
p-value	0.4047	0.0000
**Listen to Radio**		
Not listen to radio	24.4	24.0
Listen to radio	25.7	38.0
p-value	0.7634	0.0000
**Desire for children**		
Wants within 2 years	15.2	19.2
Wants after 2 years	28.8	38.3
Do not want any more	33.9	50.8
p-value	0.0010	0.0000
**Age at first marriage in years**		
<=12	34.9	28.4
13-17	23.1	37.6
18-24	27.8	34.5
25+	0.0	36.7
p-value	0.1790	0.3413
**Can refuse sex**		
No	15.4	24.7
Yes	27.0	37.6
p-value	0.0128	0.0005
**Empowerment Index**		
Low	24.8	34.2
Medium	28.2	37.7
High	22.9	35.1
p-value	0.3952	0.5942
**Visited by family planning provider In last 12 months**	26.1	35.5
No	25.3	36.0
Yes	0.8251	0.8886
p-value	1,076	1,738
Number of women (weighted)	26.1	35.5

Results reveal that, in each age group, education level, wealth index, region, residence (urban–rural), and desire for children are significantly associated with contraceptive use in 2011. The likelihood of using contraception is associated with women’s educational attainment. The more schooling a woman has, the more likely she is to report use of a modern contraceptive method. In each age group, over one-third of women with secondary or higher education, but far fewer women with no education, reported MCU. Comparing the two age groups, education seems to have a stronger effect on older women than on younger women.

Results also show that MCU is positively associated with level of household wealth. For both age groups, use of modern methods is highest among women from the richest households. Wealth-related disparities in contraceptive use are greater among younger women.

Regional variation in contraceptive use is great especially for the age group 15–24 years. This trend is observed where only 16% of the young married, sexually active women from the Northern region were using contraceptives compared to 46% from Kampala, the capital city of the country.

Women who wanted a child within two years reported the lowest levels of contraceptive use. Conversely, women who did not want any more children had the highest levels. Nevertheless, among women who did not want any more children, far fewer women age 15–24 reported current use of a contraceptive method compared with women age 24–35 (Table 2).

Women’s empowerment does not appear to be associated with contraceptive use, for either age group. Also, higher levels of contraceptive use were observed among women who said they could refuse sex with their husbands compared with women who said they could not refuse sex.

### Multivariate results

Results of the logistic regression of MCU and socio-economic characteristics are presented in Table 3. Our study carried out further analysis to find out factors that might be associated with modern contraceptive use (outcome variables), regressed over a number of independent factors with age as an interaction term. Results show that MCU varied by age and by the following factors; perception on distance to health facility, listening to radio and geographical variability. Older women had higher odds (OR = 1.97; p = 0.030) of using contraceptives compared to young ones in regard to listening to radio. Findings show that shorter distance (less than 5 kilometers) to health facility was associated with increased use of contraceptives (OR = 1.83; p = 0.02). The older women were less affected by longer distance (OR = 0.56; p = 0.013) than the young women in accessing contraceptives. Older women residing in the Eastern (OR = 3.46; p = 0.024), Northern (OR = 4.71; p = 0.021) regions had higher odds of using contraceptives compared to the young ones.

**Table 3:** **Results from logistic regression of contraception and sexually active married women’s socio-economic factors (UDHS 2011)**

Background characteristics	OR	95%CI
**Education Attainment (Reference = No education)**		
Lower Primary (P1-P3)	1.31	0.42-4.11
Upper Primary (P4-P6)	1.78	0.62-5.14
Complete Primary	1.36	0.45-4.11
Secondary	1.98	0.66-5.97
Post Secondary	12.36**	2.25-67.85
Lower Primary##Agegroup (25–34)	1.08	0.33-3.53
Upper Primary##Agegroup (25–34)	0.70	0.22-2.29
Complete Primary##Agegroup (25–34)	0.89	0.27-2.99
Secondary##Agegroup (25–34)	1.06	0.30-3.72
Post Secondary##Agegroup (25–34)	0.17	0.03-1.05
**Wealth Index (Reference = Lowest 40%)**		
Middle 40%	1.06	0.67-1.69
Highest 20%	1.40	0.80-2.47
Middle 40%##Agegroup (25–34)	1.43	0.81-2.52
Highest 20%##Agegroup (25–34)	1.65	0.77-3.52
Administrative Region (Reference = Kampala)		
Central1	0.59	0.27-1.30
Centra2	0.45	0.20-1.00
East Central	0.64	0.28-1.46
Eastern	0.32**	0.13-0.77
Northern	0.31**	0.11-0.92
Karamoja	0.63	0.06-6.47
West Nile	0.23**	0.09-0.59
Western	0.76	0.33-1.75
South Western	0.35**	0.13-0.89
Central1##Agegroup (25–34)	1.66	0.60-4.61
Centra2##Agegroup (25–34)	1.79	0.68-4.65
East Central##Agegroup (25–34)	1.17	0.42-3.28
Eastern##Agegroup (25–34)	3.38**	1.17-9.82
Northern##Agegroup (25–34)	5.02**	1.35-18.71
Karamoja##Agegroup (25–34)	1.07	0.09-12.30
West Nile##Agegroup (25–34)	3.57**	1.08-11.73
Western##Agegroup (25–34)	1.46	0.51-4.16
South Western##Agegroup (25–34)	2.67	0.89-8.02
**Perception of distance to health facility (Reference = a big problem)**		
Not a big problem	1.83**	1.26-2.67
Distance not a problem##Agegroup	0.56	0.36-0.88
Family Planning Messages		
(Reference = Did not hear family planning message On radio, TV, or newspaper in past Few months)	1.05	0.68-1.63
Heard family planning message On radio, TV, or newspaper in past Few months Heard message##Agegroup (25–34)	1.22	0.71-2.10
Listen to Radio (Reference = Not listen to radio)		
Listen to radio	0.77	0.46-1.29
Listen to radio##Agegroup (25–34)	1.97**	1.07-3.64
Desire for children (Reference = Wants within 2 years)		
Wants after 2 years	2.49**	1.54-4.03
Do not want any more	2.80	0.82-9.56
After 2 years##Agegroup (25–34)	1.18	0.64-2.17
Not any more##Agegroup (25–34)	2.03	0.51-8.04
Age at first marriage in years (Reference = <=12)		
13-17	0.56	0.23-1.36
18-24	0.54	0.22-1.31
25+	0.92	0.41-2.06
13-17##Agegroup (25–34)	2.60	0.91-7.41
18-24##Agegroup (25–34)	1.85	0.64-5.35
Can refuse sex (Reference = No)		
Yes	1.54	0.85-2.79
Yes##Agegroup (25–34)	0.94	0.48-1.82
Empowerment Index (Reference = Not empowered)		
Low	1.16	0.73-1.86
High	0.81	0.50-1.34
Low##Agegroup (25–34)	0.96	0.52-1.79
1.21 0.66-2.21	1.21	0.66-2.21
High##Agegroup (25–34)		
Visited by family planning provider (Reference = No)	0.78	0.53-1.15
Yes	1.17	0.73-1.89
Yes##Agegroup (25–34)		
Number of children living children	0.98	0.91-1.07
Number of women (weighted)	2,814	

**implies that results are significant at 5% level;

##denotes the interaction between two factors.

## Discussion

This study analyzed the socio-economic and demographic factors associated with contraceptive use in Uganda among young women (age 15–24) and older women (age 25–34) using Demographic and Health Survey data of 2011 for married, fecund, non-pregnant, and sexually active in the last year before the survey.

Results showed that listening to radio and geographical differences showed significant variability in contraceptive use among the young and the older women. Other key factors that were important for both age groups in explaining contraceptive use were; desire for children after two years and education level.

Older women who listened to radio had higher odds (1.97; p = 0.030) of using contraceptives compared to the younger women. Our study could not explicitly link radio listenership with variability among the young and older women. However, we believe that young women are more likely to be interested in non educative programmes such as secular music compared to older women who may yearn to learn more from health related talks over the radio. A number of studies that have not disaggregated radio listenership by age have shown that health related programmes including family planning are associated with contraceptive use [14].

Geographical variability was one of the factors associated with contraceptive use. The findings show that older women from the eastern and the northern regions had higher odds that is OR = 3.46; p = 0.024 and OR = 4.71; p = 0.021 respectively of using contraceptives compared to the young women. Even though the older women had higher odds of using contraceptives from both the Eastern and Northern regions, the overall odds were lowered by the young age group (East with OR = 0.31, p = 0.010; North with OR = 0.33, p = 0.049). This implies that modern contraceptive use in the two regions (Eastern and Northern) among the young women was very low. Geographical variations in contraceptive use have been found to be influenced by a number of factors and among them including community-level cultural beliefs like value attached to child [15], the presence and quality of reproductive health services [16], the physical characteristics of the area, and the presence of transport routes [17, 18]. The Northern Uganda in particular was since 1986 affected by 20 years of Lords Resistance Army rebellion which resulted into destruction of social services and breakdown of community coping mechanisms. The differentials in the Northern Uganda could be associated with increased radio programmes by a number of goverment and other organizations. As already pointed out radio programmes have influenced more of the older women than the young ones in terms of increased contraceptive use. More women from poor households in the northern and eastern Uganda may be related to early marriages that are usually associated with low rates of schooling completion and economic challenges [19].

Young age at marriage adds layers of vulnerability to women that leads to poor fertility control and fertility-related outcomes, and low maternal health care use. Our study however, found the contrary with no significant association between both age groups (young and old) and contraceptive use. This might be attributed to the fact that child bearing is expected regardless of the age at marriage as young women are expected to prove their fertility soon after marriage [5].

Our results further indicate that sexually active married women who desired to have children after two years were associated with higher odds (OR = 2.49; p = 0.000) of using modern contraceptives compared to those who wanted child bearing within two years. Some of the reasons for this postponed childbearing as stated in other studies are: women’s increased participation on the labour market, including their longer education [6, 7, 12] and career planning [6]. Furthermore, financial and practical circumstances during their studies may be difficult to combine with establishing a family, and a high educational level and a desire for career development and will increase the likelihood of delaying childbirth in women [12, 13, 20]. Young women often express a need to avoid pregnancy because they may be too young to care for a baby, they may have to end or postpone their education [1]. This might explain why the interaction term with age (young and old) were found not to be significant in the model.

As expected, education was a significant predictor in explaining variation in contraceptive use among both young and older women. Increase in education levels were significantly associated with high contraceptive use. Results also show that women who had attained post secondary level of education had very high odds (OR = 11.82; p = 0.005) of using contraceptives compared to those who had not gone to school. Education is a strong determinant of modern contraceptive utilization [21, 22] and exposes women to reproductive health information and empowers them to make appropriate decisions.

**Figure 4:**
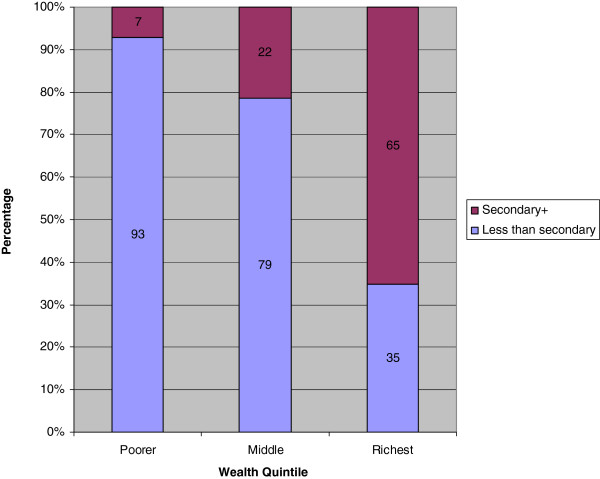
**Wealth Quintile by Education Level among married women using the UDHS 2011 data.**

Results showed that wealth was not associated with contraceptive use contrary to what would be expected. This finding might be influenced by the fact that education level appear to have influenced this relationship as more of the women (93%) with less than secondary level of education level are in the poorest wealth quintile. Comparatively, about 65% of the married women who had attained at least secondary level of education were in the richest wealth quintile (Figure 4).

Women’s empowerment has been associated with contraceptive use. However, our study results indicated that empowerment was not associated with contraceptive use contrary to what was expected. In short, empowerment did not enhance contraceptive use. This has proved to be true in certain contexts where traditional factors remain strong and empowerment’s contribution to contraceptive use is minimal [23]. The situation is not any different in Uganda where women are either under collective decision-making with their partners or completely rely on the male partner’s decision on issues that affect their reproductive life. This ultimately undermines what would have been expected to be a strong relationship between women empowerment and contraceptive use.

Even though over three-quarters (77%) of the sexually active and currently married women reported to have ever been visited by the health workers, results did not show significant association with contraceptive use. This is likely to be attributed to the fact that when women have already desired a given number of children, it might be difficult to change thier opinions. In this study, desire for children was highly associated with contraceptive use.

## Conclusions

This study has highlighted three key predictors of contraceptive use among young and older women in Uganda. First, older women who listen to radio have higher odds (OR = 1.97; p = 0.030) of using contraception compared to the younger women. Second, regarding geographical location, older women have higher odds of using contraception: particularly those from Eastern and Northern region compared to the young ones. Third, perception on distance to health facilities was a critical factor in influencing contraceptive use among the two groups.

Addressing contraceptive use among old and young women in Uganda requires concerted efforts that target such women to address the socio-economic barriers that exist. There is need for increased access of family planning service to the population through strengthening the use of Village Health Teams (VHTs) whose service is currently limited in coverage [24]. Given the variation in contraceptive use between the two age groups, our findings further suggest that there is need for variability in media targeting among the young and the older women categories for improved use of modern contraceptives, for instance using alternative media strategies to reach the young women. Family planning policies should aslo be tailored to address the specific needs of these different age groups of women with varied geographical locations.

## Abbreviations

CPR: Contraceptive prevalence rate
HIV: Human immunodeficiency virus
IUD: Intra-Uterine Device
MCU: Modern contraceptive use
OR: Odds ratio
TFR: Total fertility rate
UBOS: Uganda bureau of statistics
UDHS: Uganda Demographic and Health Survey
UNFPA: United Nations population fund
UNICEF: United Nations International Children’s Emergency Fund
VHTs: Village health teams
WHO: World Health Organization.
